# Identification of endonuclease domain-containing 1 as a novel tumor suppressor in prostate cancer

**DOI:** 10.1186/s12885-017-3330-5

**Published:** 2017-05-22

**Authors:** Jianguang Qiu, Shubin Peng, Jie Si-Tu, Cheng Hu, Wentao Huang, Yunhua Mao, Wenhan Qiu, Ke Li, Dejuan Wang

**Affiliations:** 1 Department of Urology, The Third Affiliated Hospital of Sun Yat-sen University, Guangzhou, 510630 China; 2 Department of Urology and Liver Disease Laboratory, The Third Affiliated Hospital of Sun Yat-sen University, Guangzhou, China

**Keywords:** Prostate cancer, Endonuclease domain-containing 1, Tumor suppressor

## Abstract

**Background:**

Endonuclease domain containing 1 (ENDOD1) is implicated in tumorigenesis and aggressiveness of multiple tumors. In this study, we aimed to investigate the role of ENDOD1 in prostate cancer (PCa).

**Methods:**

Immunohistochemistry were performed in 30 cases of benign prostatic hyperplasia (BPH) and 50 cases of PCa to identify its association with clinicopathological characteristics. Real-time PCR and western blot were used to detect ENDOD1 mRNA and protein expression in normal prostatic epithelial and PCa cell lines. MTT assays were employed to determine the effect of cell proliferation. Flow cytometry was used to explore the cell cycle distribution and apoptotic effects. Transwell migration and invasion assays were done to evaluate changes in the ability of cell migration and invasion.

**Results:**

Immunoreactivity scores of ENDOD1 showed no statistical difference between BPH and low-grade PCa, whereas lower immunostaining scores were observed in high-grade compared with low-grade PCa. Real-time PCR data indicated that ENDOD1 mRNA expression was markedly increased in LNCaP and 22Rv1 cells and decreased in PC3 and DU145 cells compared to the normal epithelial cells RWPE1. Western blot showed that androgen-sensitive LNCaP cells had the highest protein expression level of ENDOD1, whereas castration-resistant PCa cell lines PC3 and DU145 had significantly lower protein levels. Meanwhile, overexpression of ENDOD1 suppressed cell proliferation, induced G0/G1 cell cycle arrest and inhibited cell migration and invasion. Conversely, siRNA-mediated silencing of ENDOD1 promoted cell proliferation, migration and invasion. No apoptotic effects occurred upon manipulation of ENDOD1 expression.

**Conclusion:**

Our results indicate that ENDOD1 is a novel tumor suppressor in PCa, which may be employed as a new drug target of preventing progression to metastatic castration-resistant prostate cancer.

## Background

Prostate cancer (PCa) is the most prevalent malignant cancer and the second leading cause of cancer-related death in men in the developed countries [1]. Although the incidence of PCa in China was obviously lower than that in western developed countries, it has kept increasing greatly for the past decades [2]. Treatment approaches vary according to individual situation. The localized PCa can be cured by the radical prostatectomy or definitive radiation therapy [3, 4]. Androgen deprivation therapy (ADT) is the mainstay treatment for the recurrent and advanced PCa and initially bring great treatment benefits, but most cases eventually progress into the lethal stage which is insensitive to ADT, namely castration resistant prostate cancer (CRPC) [5, 6]. Meanwhile, metastasis often occurred in CRPC patients and is the main cause of death [7, 8]. Although studies identify many biomolecules which help uncover the underlying mechanisms of PCa progression to CRPC and metastasis, the translational applications in clinics are far from satisfaction [9, 10]. Therefore, exploring the novel biomarkers which play critical role in CRPC progression and metastasis may aid in the clinical decision-making to block disease progression, prolong patient survival and improve their quality of life.

Endonuclease domain containing 1 (ENDOD1) is a member of nucleases, which hydrolyze phosphodiester linkage in nucleic acids. It has been reported that nucleases participate in mutation avoidance, DNA repair and programmed cell death [11–13]. In addition, ENDOD1 belongs to the subgroup of non-sugar specific nuclease which hydrolyze both DNA and RNA without any apparent base preference [14]. Aberrant expression of ENDOD1 has been observed in several types of tumors. In soft tissue tumors, decreased ENDOD1 correlated with local aggressiveness [15]. Similarly, downregulation of ENDOD1 was revealed in colorectal cancer compared to the normal mucosa and proposed to be involved in epithelial tumorigenesis [16]. Moreover, quantitative real-time polymerase-chain-reaction (RT-PCR) analysis revealed a decreased mRNA expression of ENDOD1 in high-grade and lymphnode-metastatic PCa [17]. Nevertheless, the biological function of ENDOD1 in PCa remains to be clarified.

In this study, we determined the protein expression of ENDOD1 in PCa tissues by immunohistochemistry (IHC) and explored its correlations with clinicopathological parameters. We for the first time investigated the role of ENODD1 in cell proliferation, apoptosis, migration and invasion in PCa cells. Our findings showed that ENDOD1 protein expression exhibited a significant down-regulation in high-grade PCa tissues. In vitro studies indicated ENDOD1 inhibited cell proliferation, migration and invasion in PCa cells.

## Methods

### Human prostatic tissues and immunohistochemistry (IHC)

We examined a total of 30 BPH samples and 50 PCa samples in this study. The diagnosis was confirmed histologically after surgery. The BPH tissues and PCa tissues were respectively from patients treated by transurethral resection of prostate and laparoscopic radical prostatectomy in the Third Affiliated Hospital of Sun Yat-sen University during 1st January 2012 and 31st December 2015. IHC was performed as described previously [18]. Briefly, tissues were deparaffinized and then antigen retrieval was done in 10 mmol/L citrate buffer (pH 6.0) for 15 min at 100 °C. After blocking endogenous peroxidase with methanol containing 0.3% hydrogen peroxide (H_2_O_2_) for 15 mins, tissue sections were incubated overnight at 4 °C using a rabbit polyclonal ENDOD1 antibody (Abcam, ab121293, 1:500). All immunostained sections were reviewed by two pathologists independently who were blinded to the patients’ clinicopathological information. The analysis of immunostaining intensity was acquired by multiplying the level of staining intensity (negative = 0, weak = 1, moderate = 2, strong = 3) and percentage of positively stained cells (0% = 0, <10% = 1, 11–50% = 2, 51–80% = 3, >80% = 4), as described previously [19]. This study was approved by our Institutional Ethics Committee and all patients had signed the informed consents for using their surgically-resected tissues and related information.

### Cell lines and cell culture

Human prostatic cancer cell lines DU145 (HTB-81™), PC3 (CRL-1435™), LNCaP (CRL-1740™) and 22Rv1 (CRL-2505™) and the non-malignant human prostatic normal epithelial cell RWPE-1 (CRL-11609™) were obtained from the American Type Culture Collection (ATCC) and routinely cultured in RPMI1640 medium (GIBCO, USA) supplemented with 10% fetal bovine serum (FBS) in a humidified 37 °C incubator containing 5% CO_2_, according to the ATCC instructions.

### RNA isolation and qPCR

Total RNA was extracted from RWPE-1, DU145, PC3, LNCaP and 22Rv1 using Trizol reagent (Invitrogen Life Technologies, CA, USA). cDNA was synthesized using 2 μg total RNA and SYBR® premix Ex Taq II (Takara, Japan). qPCR was performed on the ABI Prism 7500 fast Sequence Detection System (Applied Biosystem). The 2^-ΔΔCt^ method was performed to analyze relative quantities for the level of gene expression. The following primers were used: GAPDH forward 5′-TCCTCTGACTTCAACAGCGACACC-3’and reverse 5′-TCTCTCTTCCTCTTGTGCTCTTGG-3′, ENDOD1 forward 5′-GACCGCATCCCCGTGTA-3′ and reverse 5′-AATCGCCTCCTCAAGGTT-3′.

### Transfection with expression vectors and small interfering RNA (siRNA)

The ENDOD1-expressing construct pCMV-N-Flag-ENDOD1 was performed as previously reported [20]. Genomic cDNA from PC3 cells was used as templates to amplify the upper and lower genes with the sites of EcoR I and Xho I restriction enzymes. Taq enzyme was used to obtain amplifications. After digested by the Xho I and EcoR I restriction enzymes, ENDOD1 and pCMV-N-Flag plasmid were extracted and purified using Gel Extraction Kit (Omega biotech) according to the manufacturer’s instructions. Then products were mixed, connected with DNA Ligation Kit (Takara) and transformed into DH5α peptide (Takara). After coated the lysogeny broth solid medium with kanamycin 100 μg/ml for 16 h, single colonies were chosen randomly. Colonies were inoculated in the lysogeny broth medium containing with kanamycin 100 μg/ml overnight at 37 °C, 150 r/min and then plasmid DNA was extracted according to instructions of Endo-Free Plasmid Midi Kit (Omega biotech). The plasmids were sequenced before transfection (BGI tech, China). Cells were transfected using Lipofectamine 2000 (Invitrogen) according to the manufacturer’s instructions. Cells were also transfected with siRNA specific to ENDOD1 (5′-CAGAUUACCUUGAUUCUGATT-3′) (GenePharma, Shanghai, China) using Lipofectamine 2000 (Invitrogen), following the manufacturer’s instructions. The concentration of siRNA used was 100 nM. After 6 h of transfection, the complete culture media was added. The assays were done 48 h post-transfection.

### Cell growth assay

The DU145 with overexpression and negative control were cultured in 96-well culture plates at the number of 3000 cells per well, in RPMI-1640 medium containing 10% FBS. LNCaP transfected with siRNA and si-NC were cultured at a cell density of 5000 cells/well under the same conditions as DU145. The growth of the cells was analyzed by MTT assay (KeyGEN BioTECH, Nanjing, China). Optical density (OD), which was detected by an Automatic microplatereader (TEAN, Swiss) at a 490 nm wave length. The rate of cell proliferation was calculated. Three independent experiments were performed over multiple days.

### Flow cytometry (FCM)

Cell cycle and apoptosis studies were performed using FCM on DU145 transfected with ENDOD1 overexpression and the control plasmids, LNCaP transfected with siRNA and si-NC, respectively. Cells were seeded at a concentration of 3 × 10^6^ cells/mL. For cell cycle analysis, the cells were dispersed in 500 μL PBS (1%, pH 7) with PI (50 μg/mL) and RNase A (100 μg/mL) and kept in dark for 15 min before FCM analysis. For apoptosis study, cells were treated with PI (50 μg/mL), annexin V-FITC conjugate (5 μg/mL)(KeyGEN BioTECH, Nanjing, China) and dispersed in PBS (1%, pH 7). Each analysis was performed at least thrice (*n* = 3) and a count of a minimum of 10,000 events was taken for each analysis. Data are represented as mean ± S.D.

### In vitro migration and invasion assays

The migration and invasion activity of DU145 cells with overexpression ENDOD1 and LNCaP cells with siRNA were assessed using transwell migration assays and matrigel invasion assays as previously described [21]. Cells were seeded into the upper chamber of the transwell in 24-well plates (membrane pore size, 8 μm; Corning Incorporated; Corning, NY, USA) with Matrigel (BD Pharmingen), and 500 μl RPMI-1640 medium with 15% FBS was added into the lower chamber. After 24 h, the bottoms of the inserts were fixed in paraformaldehyde solution for 10 mins and stained with 0.1% crystal violet staining solution. The cells invading into the bottom-lower surfaces of inserts were measured by using microscope at ×100. Results were repeated in triplicate.

### Western blot analysis

The western blot was performed as previously reported [22]. Cells were lysed by using RIPA buffer containing 1% PMSF. Protein concentration was quantified with the BCA Protein Kit (KeyGEN BioTECH, Nanjing, China) and equal amounts of proteins were loaded and separated by 12% SDS-PAGE, then transferred a to a PVDF membrane (Millpore, USA) and probed with primary antibody against ENDOD1 (Abcam, ab121293,1:2000) using antibodies against ENDOD1 (Abcam, ab121293, 1:2000). All the levels of protein were normalized to GAPDH (Abcam, ab9485,1:2500).

### Statistical analysis

Data are expressed as the means ± SD. Data significance was analyzed using Student’s t-test, Mann–Whitney U-test and Chi-square χ2 tests. A value of *P* < 0.05 was considered to indicate a statistically significant difference. All analyses were performed with SPSS version 19.0 (SPSS Inc., Chicago, IL, USA).

## Results

### ENDOD1 immunostaining and correlation with clinicopathalogical factors of PCa

**Fig. 1:**
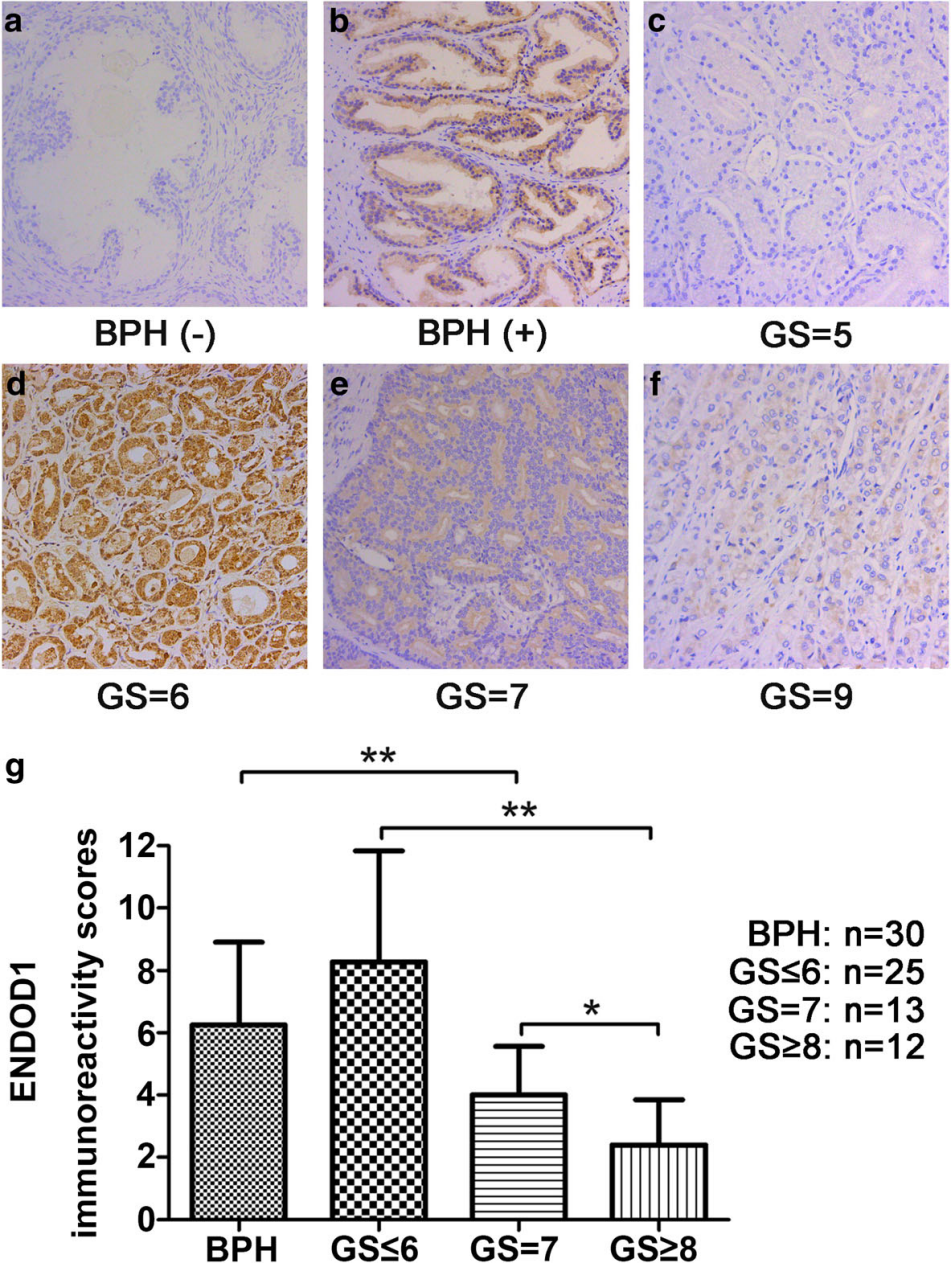
ENDOD1 is downregulated in PCa tissues with high Gleason scores. **a**-**f** Representative images showed the ENDOD1 immunostaining intensity. BPH tissues showed negative (**a**) and moderate (**b**) ENDOD1 staining. PCa tissues showed negative (**c**), strong (**d**), and moderate (**e**) and weak (**f**) ENDOD1 staining, respectively. Magnification 200×. **g**, Immunoreactivity scores (IRS) analysis of ENDOD1 in BPH tissues and PCa tissues. IRS were determined by multiplying the level of staining intensity (negative = 0, weak = 1, moderate = 2, strong = 3) and percentage of positively stained cells (0% = 0, <10% = 1, 11–50% = 2, 51–80% = 3, >80% = 4). Results indicated that PCa tissues with higher Gleason scores (Gleason score ≥ 7) showed significantly lower ENDOD1 expression than that with low Gleason score and BPH tissues. *P<0.05; **P<0.01

We detected ENDOD1 protein expression in 30 BPH patients and 50 PCa patients by IHC. The mean ages of BPH patients and PCa patients were 54.2 (45–84) years and 68.3 (50–82) years, respectively. The average PSA value was 9.4 (2.1–25.6) ng/mL in BPH patients and 24.2 (3.8–175.5) ng/mL in PCa patients. The basic clinical characteristics of PCa patients are summarized in Table 1. No association was found between ENDOD1 expression and age (*P* = 0.749), preoperative PSA (*P* = 0.196), clinical T stage (*P* = 0.443), biopsy Gleason score (*P* = 0.191), pathological T stage (*P* = 0.292), seminal vesicle invasion (*P* = 0.139), lymphnode involvement (*P* = 0.064). While a significant difference was revealed between ENDOD1 expression and postoperative Gleason score (*P* = 0.011) and surgical margin status (*P* = 0.024). Representative images of immunostaining in BPH tissues and PCa tissues are shown in Fig. 1a-f. Immunoreactivity score analysis demonstrated that there was no difference in ENDOD1 expression between BPH samples and PCa tissues with low Gleason score (Gleason score < 7) (Fig. 1g). However, PCa tissues with high Gleason score (Gleason score ≥ 7) displayed significantly lower ENDOD1 expression than that with low Gleason score and BPH (Fig. 1g). These findings suggested that ENDOD1 down regulation might play a role in the progression of PCa.

**Table 1:** Patients charateristics and ENDOD1 expression of 50 prostate cancer patients

	Total no. cases (%)	ENDOD1 expression		*P*-value
		Low or none no. cases (%)	High no. cases (%)	
All cases	50 (100)	22 (100)	28 (100)	
Age				
<69	24 (48)	10 (45.5)	14 (50)	0.749
≥69	26 (52)	12 (54.5)	14 (50)	
PSA (ng/ml)				
<10	25 (50)	8 (36.4)	17 (60.7)	0.196
10–20	12 (24)	6 (27.2)	6 (21.4)	
>20	13(26)	8(36.4)	5(17.9)	
Clinical T stage				
cT1	37(74)	15(68.2)	22(78.6)	0.443
cT2	12(24)	6(27.3)	6(21.4)	
cT3	1(2)	1(4.5)	0(0)	
Biopsy Gleason score				
≤6	25(50)	9(40.9)	16(57.1)	0.191
7	13(24)	5(22.7)	8(28.6)	
8–10	12(26)	8(36.4)	4(14.3)	
Pathological T stage				
pT2	33(66)	12(54.5)	21(75)	0.292
pT3a	4(8)	2(9.1)	2(7.1)	
pT3b	13(26)	8(36.4)	5(17.9)	
Post-op Gleason score				
≤6	22(44)	6(27.3)	16(57.1)	0.011
7	10(20)	3(13.6)	7(25)	
8–10	18(36)	13(59.1)	5(17.9)	
Seminal vesicle invasion				
Yes	13(26)	8(36.4)	5(17.9)	0.139
No	37(74)	14(63.6)	23(82.1)	
Lymphnode involvement				
Yes	10(20)	7(31.8)	3(10.7)	0.064
No	40(80)	15(68.2)	25(89.3)	
Surgical margin status				
Yes	9(18)	7(31.8)	2(7.2)	0.024
No	41(82)	15(68.2)	26(92.9)

### ENDOD1 expression in PCa cell lines

**Fig. 2:**
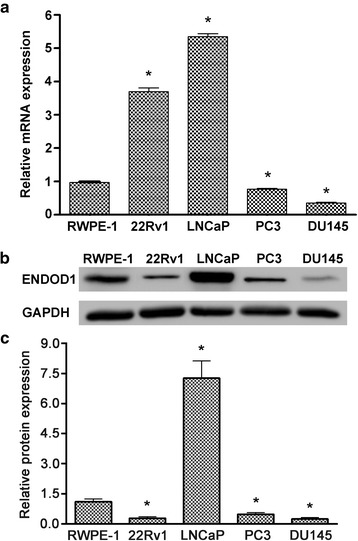
ENDOD1 expression in prostate cancer cell lines. **a** ENDOD1 and GAPDH mRNA were determined by quantitative PCR in immortalized epithelial cells (RWPE-1) and PCa cells (22Rv1, LNCaP, PC3 and DU145). Data were represented as relative expression (mean ± S.E.M.) standardized by endogenous expression of GAPDH. *P < 0.05. **b**-**c** The cell lysates were immunoblotted with anti-ENDOD1 and anti-GAPDH antibodies, respectively. Experiments were performed at least three times in triplicate

In order to investigate the expression of ENDOD1 in PCa cells, Real-time PCR analysis of ENDOD1 mRNA expression was performed in a panel of normal prostatic epithelial and PCa cell lines (22Rv1, LNCaP, PC3 and DU145). The data indicated that the level of ENDOD1 expression was markedly increased in LNCaP and 22Rv1 cells lines (mean ± SD, *n* = 3, *P* < 0.05) and decreased in PC3 and DU145 (mean ± SD, *n* = 3, *P* < 0.05) compared to the normal epithelial cell RWPE1 (Fig. 2a). Western blot showed that androgen-sensitive LNCaP cells had the highest protein expression level of ENDOD1, whereas castration resistant PCa cell lines PC3 and DU145 had significantly lower levels of ENDOD1 (Fig. 2b and c). However, the protein level is not proportional to the mRNA level in 22RV1 (Fig. 2b and c). These results indicated that ENDOD1 may be involved in the progression from androgen dependence (AD) to androgen independence (AI). For further investigation on the impact of ENDOD1 in tumor biology of PCa, we selected LNCaP and DU145 cells as study models.

### ENDOD1 suppresses the proliferation of PCa cells

**Fig. 3:**
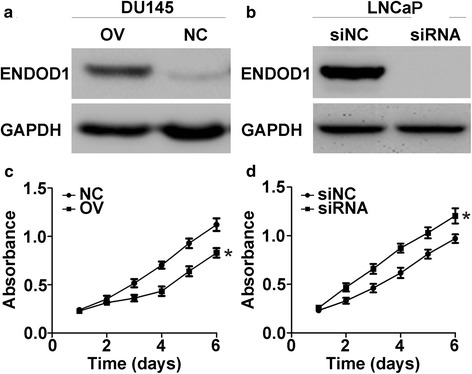
ENDOD1 suppresses the proliferation of prostate cancer cells. **a**-**b** Western blot analysis confirmed ENDOD1 overexpression in DU145 (**a**) and downregulation in LNCaP (**b**) cells. **c**-**d** MTT assays demonstrated inhibitory effect of ENDOD1 on cell proliferation of PCa cells. ENDOD1 overexpression significantly suppressed proliferation of DU145 cells (**c**), whereas siRNA-induced downregulation of ENDON1promoted proliferation of LNCaP cells (**d**). Cell growth curves were plotted according to absorbance at 490 nm. *P < 0.05

To explore the effect of ENDOD1 on proliferation of PCa cells, eukaryotic overexpression plasmid pCMV-N-Flag-ENDOD1 and specific small siRNA was transfected into DU145 and LNCaP cells respectively. The expression of ENDOD1 was confirmed by western blot (Fig. 3a and b). We next investigated the cell proliferation rate of these cells by MTT assay. After transfection with ENDOD1, DU145 cells showed significantly decreased OD values in 96 h post-transfection, suggesting an inhibitory effect of ENDOD1 on proliferation of DU145 (*P* < 0.05, Fig. 3c). Conversely, LNCaP cells proliferated remarkably faster after siRNA-mediated down regulation of ENDOD1 (*P* < 0.05, Fig. 3d). Therefore, ENDOD1 suppresses cell proliferation in PCa cells.

### Effects of ENDOD1 on cell cycle and apoptosis

**Fig. 4:**
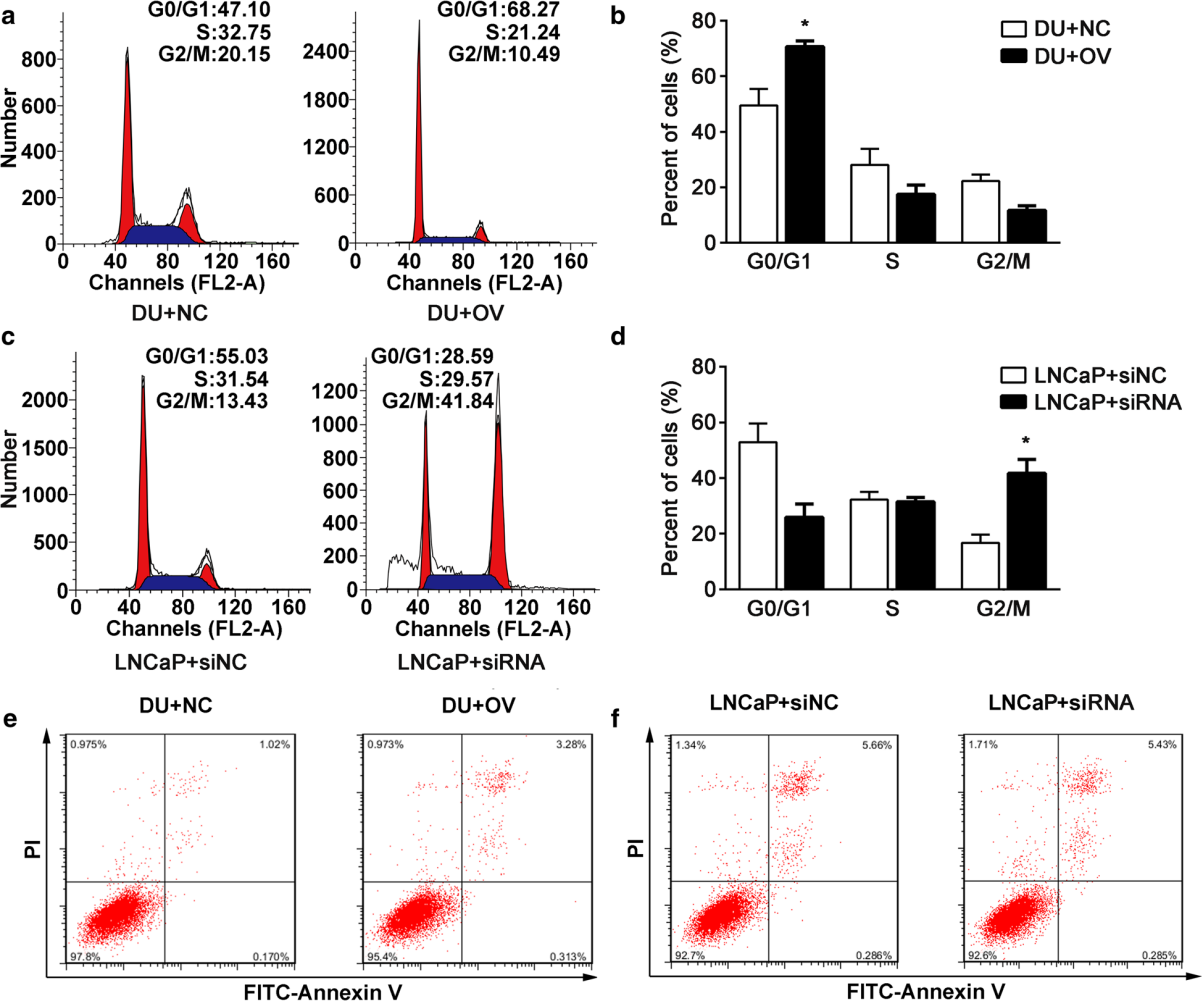
Effects of ENDOD1 on cell cycle and apoptosis. **a**-**b** cell cycle distribution without or with overexpression of ENDOD1 in DU145 cells. Overexpression of ENDOD1 led to notable increases in G0/G1 phase arrest, with a corresponding decrease in the percentage of cells in the G2/M and S phases. **c**-**d** cell cycle distribution after transfecting siRNA or si-NC of ENDOD1 in LNCaP cells. siRNA-induced downregulation of ENDON1 led to remarkable increase in the percentage of cells in the G2/M phase. **e**-**f** FITC-Anexin V test demonstrated no changes in apoptosis between the control group and the group of ENDOD1 overexpression (**e**) or siRNA (**f**). Error bars represent the means of three independent experiments. *P <0.05.

To identify changes in cell cycle distribution, we performed FCM after manipulation of ENDOD1 expression. Overexpression of ENDOD1 in DU145 cells led to notable increases in G0/G1 phase arrest, with a corresponding decrease in the percentage of cells in the G2/M and S phases, compared with control group (Fig. 4a and b, *P* <0.01). LNCaP cells transfected with siRNA reversed the effect, with a remarkable increase in the percentage of cells in the G2/M phase (Fig. 4c and d, *P* <0.01). Moreover, we measured cell apoptotic rate to determine whether ENDOD1 promotes apoptosis. As shown in Fig. 4e and f, the percentage of apoptotic cells were 3.28% vs 1.02% for DU145 cells with ENDOD1 expression and the control, respectively and were 5.53% vs 5.66% for LNCaP cells with siRNA and si-NC, respectively, showing no statistical difference. Collectively, these data suggest that ENDOD1 inhibits cell cycle progression at the G0/G1 arrest and has no effect on cell apoptosis.

### ENDOD1 inhibits migration and invasion in PCa cells

**Fig. 5:**
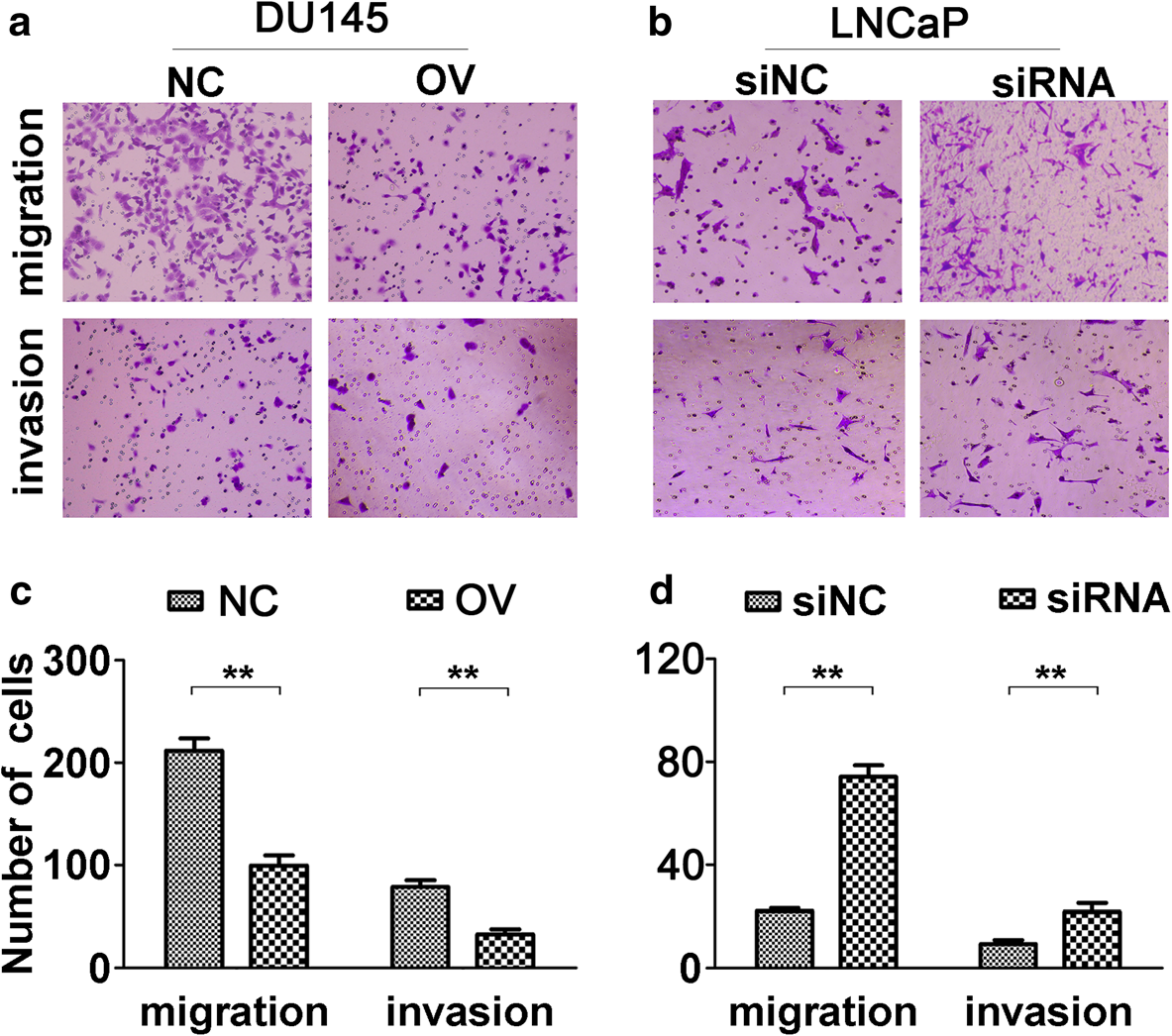
ENDOD1 inhibits migration and invasion in PCa cells. **a** and **c** Overexpression of ENDOD1 inhibited migration and invasion in DU145 cells. **b** and **d** Silencing of ENDOD1 promoted migration and invasion in LNCaP cells. Representative images were shown in **a** and **b**, and cell numbers of migrated or invaded through chamber membrane were analyzed in **c** and **d**. The data were presented as the mean ± S.D. of three independent experiments. *P <0.05, **P <0.01

As for the role of ENDOD1 in migration and invasion of PCa cells, we performed transwell migration and invasion assays. ENDOD1 overexpression significantly suppressed cell migration and invasion in DU145 (Fig. 5a and c) whereas knockdown of ENDOD1 by siRNA promoted cell migration and invasion in LNCaP cells (Fig. 5b and d). Taken together, there is an obvious negative regulation of migration and invasion by ENDOD1 expression in PCa cells.

## Discussion

Metastatic castration-resistant prostate cancer (mCRPC) is the lethal and incurable stage of PCa. Currently, the overall survival of patients with mCRPC is about 40.7 months regardless of multidisciplinary treatment modality [23]. Researches on the mechanisms whereby PCa progresses to mCRPC identify large numbers of oncotargets, but few translate into clinical implication [6, 24]. Therefore, any efforts on discovering biomarkers that involve in PCa progression are worthwhile and may bring breakthrough in target-directed treatments in PCa.

It is reported that endonuclease domain-containing 1 (ENDOD1) is aberrantly deregulated in several tumors. Gaedcke J et al. proposed that ENDOD1 involved in epithelial tumorigenesis of colorectal cancer and could be used as a novel tumor marker [16]. Our results didn’t showed differences in ENDOD1 expression in tissues between BPH group and low-grade PCa group whereas significant downregulation was observed in high-grade PCa group, suggesting ENDOD1 may exert aggressiveness-promoting function in PCa. Cunha IW et al. revealed inverse correlation between ENDOD1 expression and local aggressiveness in soft tissue tumors and reported that ENDOD1 may play a role in tumor biology [15]. Consistently, we demonstrated that the ENDOD1 expression in PCa tissues was negatively correlated with Gleason score which is widely considered as a marker of aggressiveness. These results indicate a negative role of ENDOD1 in tumor progression.

In PCa cell lines, ENDOD1 expression in mCRPC cells were significantly decreased compared with androgen-sensitive LNCaP cells, suggesting ENDOD1 may promote PCa progression to CRPC. Marques RB et al. demonstrated that ENDOD1 is regulated genes of androgen receptor (AR) in hormonal therapy-resistant PCa cells [17]. Since AR plays critical role in promoting PCa tumor growth and progression [25], ENDOD1 is likely to be the mediator of AR signaling in the progression to mCRPC and could possibly be used as markers in predicting the course of disease. It should be noted that the protein level is not proportional to the mRNA of ENDOD1 in 22RV1 cells. This could be caused by a multitude of post-transcriptional mechanisms such as mRNA and protein degradation [26], but the specific mechanisms need further investigation.

Moreover, we found that ENDOD1 inhibited cell proliferation, migration and invasion. Of literatures, ENDOD1 is a member of the DNA/RNA sugar non-specific nucleases [14]. Nucleases are implicated in mutation avoidance, DNA repair and programmed cell death via hydrolyzing phosphodiester linkage in nucleic acids [11–13]. Aberrant alteration of mutation, DNA repair and programmed cell death often occurred in PCa and were considered to associate with cancer predispositions and progression [27–29]. Therefore, there’s probability that ENDOD1 functions as tumor suppressor through preventing aberrant alteration of mutation, DNA repair and programmed cell death. However, it needs further investigation to elucidate the specific mechanisms.

There are some limitations in our study. Firstly, the number of PCa patients is relatively small, but it is sufficient to guarantee the validity of statistical significance. Secondly, follow-up data for the PCa patients hasn’t been analyzed to explore the prognostic value of ENDOD1 in PCa. Thirdly, the specific mechanisms whereby ENDOD1 exert inhibitory activity remain elusive. Nevertheless, our study identified ENDOD1 is a novel tumor suppressor, which may add our knowledge about PCa progression.

## Conclusion

In this study, we have identified ENDOD1 as a novel tumor suppressor in PCa. ENDOD1 may be employed as drug target to prevent disease progression to mCRPC.

## Abbreviations

AD: androgen dependence
ADT: androgen deprivation therapy
AI: androgen independence
ATCC: American Type Culture Collection
BPH: benign prostatic hyperplasia
CRPC: castration resistant prostate cancer
ENDOD1: endonuclease domain containing 1
FBS: fetal bovine serum
FCM: flow cytometry,
IHC: immunohistochemistry
mCRPC: metastatic castration-resistant prostate cancer
OD: optical density
PCa: prostate cancer
siRNA: small interfering RNA

## References

[1] Siegel RL, Miller KD, Jemal A (2015). Cancer statistics, 2015. CA Cancer J Clin.

[2] Chen W, Zheng R, Baade PD, Zhang S, Zeng H, Bray F, Jemal A, Yu XQ, He J (2016). Cancer statistics in China, 2015. CA Cancer J Clin.

[3] Hamdy FC, Donovan JL, Lane JA, Mason M, Metcalfe C, Holding P, Davis M, Peters TJ, Turner EL, Martin RM (2016). 10-year outcomes after monitoring, surgery, or radiotherapy for localized prostate cancer. N Engl J Med.

[4] Keyes M, Crook J, Morton G, Vigneault E, Usmani N, Morris WJ (2013). Treatment options for localized prostate cancer. Canadian family physician Medecin de famille canadien.

[5] Wang Q, Li W, Zhang Y, Yuan X, Xu K, Yu J, Chen Z, Beroukhim R, Wang H, Lupien M (2009). Androgen receptor regulates a distinct transcription program in androgen-independent prostate cancer. Cell.

[6] Watson PA, Arora VK, Sawyers CL (2015). Emerging mechanisms of resistance to androgen receptor inhibitors in prostate cancer. Nat Rev Cancer.

[7] Robinson D, Van Allen EM, Wu YM, Schultz N, Lonigro RJ, Mosquera JM, Montgomery B, Taplin ME, Pritchard CC, Attard G (2015). Integrative clinical genomics of advanced prostate cancer. Cell.

[8] Gartrell BA, Coleman R, Efstathiou E, Fizazi K, Logothetis CJ, Smith MR, Sonpavde G, Sartor O, Saad F (2015). Metastatic prostate cancer and the bone: significance and therapeutic options. Eur Urol.

[9] Hsieh CL, Botta G, Gao S, Li T, Van Allen EM, Treacy DJ, Cai C, He HH, Sweeney CJ, Brown M (2015). PLZF, a tumor suppressor genetically lost in metastatic castration-resistant prostate cancer, is a mediator of resistance to androgen deprivation therapy. Cancer Res.

[10] Roychowdhury S, Chinnaiyan AM (2013). Advancing precision medicine for prostate cancer through genomics. Journal of clinical oncology : official journal of the American Society of Clinical Oncology.

[11] Sun X, Thrower D, Qiu J, Wu P, Zheng L, Zhou M, Bachant J, Wilson DM, Shen B (2003). Complementary functions of the *Saccharomyces cerevisiae* Rad2 family nucleases in Okazaki fragment maturation, mutation avoidance, and chromosome stability. DNA Repair.

[12] Doherty R, Madhusudan S (2015). DNA repair endonucleases: physiological roles and potential as drug targets. J Biomol Screen.

[13] Kawane K, Nagata S (2008). Nucleases in programmed cell death. Methods Enzymol.

[14] Lyu ZZ, Zhao BB, Koiwai K, Hirono I, Kondo H (2016). Identification of endonuclease domain-containing 1 gene in Japanese flounder *Paralichthys olivaceus*. Fish & shellfish immunology.

[15] Cunha IW, Carvalho KC, Martins WK, Marques SM, Muto NH, Falzoni R, Rocha RM, Aguiar S, Simoes ACQ, Fahham L (2010). Identification of genes associated with local aggressiveness and metastatic behavior in soft tissue tumors. Transl Oncol.

[16] Gaedcke J, Grade M, Jung K, Camps J, Jo P, Emons G, Gehoff A, Sax U, Schirmer M, Becker H (2010). Mutated KRAS results in overexpression of DUSP4, a MAP-kinase phosphatase, and SMYD3, a histone methyltransferase, in rectal carcinomas. Genes, chromosomes & cancer.

[17] Marques RB, Dits NF, Erkens-Schulze S, van Ijcken WF, van Weerden WM, Jenster G (2011). Modulation of androgen receptor signaling in hormonal therapy-resistant prostate cancer cell lines. PLoS One.

[18] Mao Y, Li K, Lu L, Si-Tu J, Lu M, Gao X (2016). Overexpression of Cdc20 in clinically localized prostate cancer: relation to high Gleason score and biochemical recurrence after laparoscopic radical prostatectomy. Cancer biomarkers : section A of Disease markers.

[19] Yu S, Xu Z, Zou C, Wu D, Wang Y, Yao X, Ng CF, Chan FL (2014). Ion channel TRPM8 promotes hypoxic growth of prostate cancer cells via an O2 -independent and RACK1-mediated mechanism of HIF-1alpha stabilization. J Pathol.

[20] Cai G, Wu D, Wang Z, Xu Z, Wong KB, Ng CF, Chan FL, Yu S. Collapsin response mediator protein-1 (CRMP1) acts as an invasion and metastasis suppressor of prostate cancer via its suppression of epithelial-mesenchymal transition and remodeling of actin cytoskeleton organization. Oncogene. 2016; 10.1038/onc.2016.227 PMC5290039 27321179

[21] Chu JH, Yu S, Hayward SW, Chan FL (2009). Development of a three-dimensional culture model of prostatic epithelial cells and its use for the study of epithelial-mesenchymal transition and inhibition of PI3K pathway in prostate cancer. Prostate.

[22] Li K, Pang J, Cheng H, Liu WP, Di JM, Xiao HJ, Luo Y, Zhang H, Huang WT, Chen MK (2015). Manipulation of prostate cancer metastasis by locus-specific modification of the CRMP4 promoter region using chimeric TALE DNA methyltransferase and demethylase. Oncotarget.

[23] Omlin A, Pezaro C, Mukherji D, Mulick Cassidy A, Sandhu S, Bianchini D, Olmos D, Ferraldeschi R, Maier G, Thompson E (2013). Improved survival in a cohort of trial participants with metastatic castration-resistant prostate cancer demonstrates the need for updated prognostic nomograms. Eur Urol.

[24] Agarwal N, Sonpavde G, Sternberg CN (2012). Novel molecular targets for the therapy of castration-resistant prostate cancer. Eur Urol.

[25] Mills IG (2014). Maintaining and reprogramming genomic androgen receptor activity in prostate cancer. Nat Rev Cancer.

[26] Vogel C, Abreu Rde S, Ko D, Le SY, Shapiro BA, Burns SC, Sandhu D, Boutz DR, Marcotte EM, Penalva LO (2010). Sequence signatures and mRNA concentration can explain two-thirds of protein abundance variation in a human cell line. Mol Syst Biol.

[27] Goodall ML, Fitzwalter BE, Zahedi S, Wu M, Rodriguez D, Mulcahy-Levy JM, Green DR, Morgan M, Cramer SD, Thorburn A (2016). The autophagy machinery controls cell death switching between apoptosis and necroptosis. Dev Cell.

[28] Mateo J, Boysen G, Barbieri CE, Bryant HE, Castro E, Nelson PS, Olmos D, Pritchard CC, Rubin MA, de Bono JS. DNA repair in prostate cancer: biology and clinical implications. Eur Urol. 2016; 10.1016/j.eururo.2016.08.037 27590317

[29] Chang KH, Li R, Kuri B, Lotan Y, Roehrborn CG, Liu J, Vessella R, Nelson PS, Kapur P, Guo X (2013). A gain-of-function mutation in DHT synthesis in castration-resistant prostate cancer. Cell.

