# Erratum to: Identification of endonuclease domain-containing 1 as a novel tumor suppressor in prostate cancer

**DOI:** 10.1186/s12885-017-3517-9

**Published:** 2017-08-24

**Authors:** Jianguang Qiu, Shubin Peng, Jie Si-Tu, Cheng Hu, Wentao Huang, Yunhua Mao, Wenhan Qiu, Ke Li, Dejuan Wang

**Affiliations:** 1 Department of Urology, The Third Affiliated Hospital of Sun Yat-sen University, Guangzhou, 510630 China; 2 Department of Urology and Liver Disease Laboratory, The Third Affiliated Hospital of Sun Yat-sen University, Guangzhou, China

## Erratum

After publication of the original article [1] the authors found that the case number “n” had been incorrectly marked for each Gleason group in Figure 1g (Fig. 1g).

**Fig. 1:**
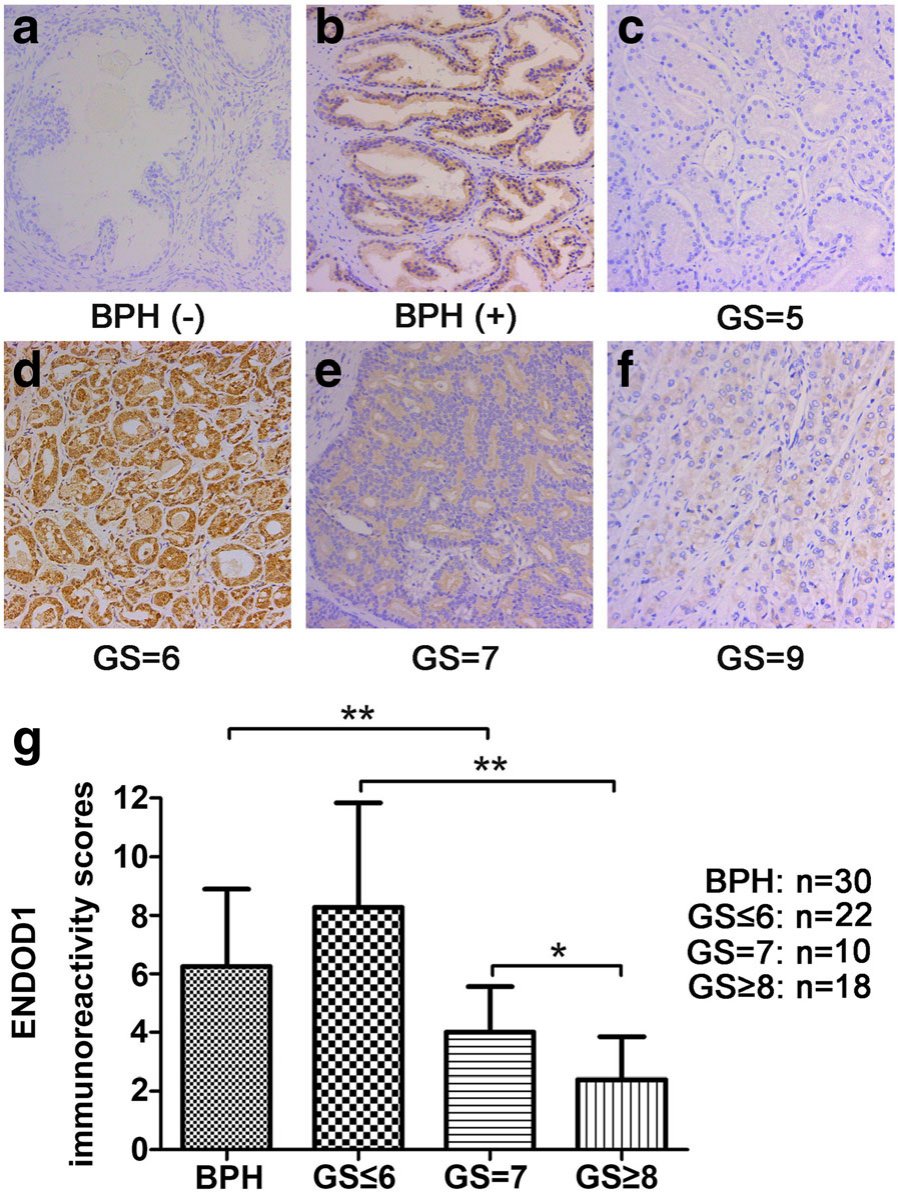
ENDOD1 is downregulated in PCa tissues with high Gleason scores. **a**-**f** Representative images showed the ENDOD1 immunostaining intensity. BPH tissues showed negative (**a**) and moderate (**b**) ENDOD1 staining. PCa tissues showed negative (**c**), strong (**d**), and moderate (**e**) and weak (**f**) ENDOD1 staining, respectively. Magnification 200×. **g**, Immunoreactivity scores (IRS) analysis of ENDOD1 in BPH tissues and PCa tissues. IRS were determined by multiplying the level of staining intensity (negative = 0, weak = 1, moderate = 2, strong = 3) and percentage of positively stained cells (0% = 0, <10% = 1, 11–50% = 2, 51–80% = 3, >80% = 4). Results indicated that PCa tissues with higher Gleason scores (Gleason score ≥ 7) showed significantly lower ENDOD1 expression than that with low Gleason score and BPH tissues. *P<0.05; **P<0.01

The biopsy Gleason group information had been used in the original article:

BPH: n=30

GS≤6: n=25

GS=7: n=13

GS≥8: n=12

Data should instead have been taken from the post-operative Gleason group, and the figures should therefore appear as the following in Figure 1g:

BPH: n=30

GS≤6: n=22

GS=7: n=10

GS≥8: n=18

A corrected, full version of Figure 1 with the above amendments has been included in this Erratum.

Corrected Figure 1

## References

[1] Qiu J, Peng S, Si-Tu J, Hu C, Huang W, Mao Y, Qui W, Li K, Wang D (2017). Identification of endonuclease domain-containing 1 as a novel tumor suppressor in prostate cancer. BMC Cancer.

